# The Protective Effects of *α*B-Crystallin on Ischemia-Reperfusion Injury in the Rat Retina

**DOI:** 10.1155/2017/7205408

**Published:** 2017-10-02

**Authors:** Huan Yan, Yanli Peng, Wei Huang, Liyan Gong, Li Li

**Affiliations:** ^1^Department of Ophthalmology, The Second Affiliated Hospital of Chongqing Medical University, Chongqing 400010, China; ^2^Chongqing Aier-Mega Eye Hospital, Aier Eye Hospital Group, Chongqing 400060, China

## Abstract

To investigate whether *α*B-crystallin protects against acute retinal ischemic reperfusion injury (I/R) and elucidate the potential antioxidant mechanisms. Retinal I/R injury was made by elevating the intraocular pressure (IOP) 110 mmHg for 60 min, and *α*B-crystallin (1 × 10^−5^ g/L) or vehicle solution was administered intravitreously immediately after I/R injury. The animal was sacrificed 24 h, 1 w, and 1 m after the I/R injury. The retina damage was detected by hematoxylin and eosin (HE) staining and electroretinography (ERG). The level of malondialdehyde (MDA), nitric oxide (NO), and the total superoxide dismutase (T-SOD) was determined. An immunohistochemical study was performed to detect the activation of inducible nitric oxide synthase (iNOS) and NF- (nuclear factor-) kappaB (NF-*κ*B) p65. The decrease of retinal thickness and the number of retinal ganglion cells (RGCs) can be suppressed by *α*B-crystallin. And the amplitudes of a- and b-wave were remarkably greater without *α*B-crystallin. Similarly, *α*B-crystallin also significantly decreased the level of MDA and NO and enhanced the activities of T-SOD. The positive expression of iNOS and NF-kappaB p65 was obviously reduced while treated with *α*B-crystallin. *α*B-crystallin can inhibit the expression of NF-*κ*B and its antioxidative effect to protect the retina from I/R injury.

## 1. Introduction

Retinal ischemia reperfusion (I/R) injury is responsible for several vision-threatening ocular diseases such as retinal vascular occlusion, acute glaucoma, diabetic retinopathy, and retinopathy of prematurity [1, 2]. Pathological changes are involved in I/R, including energy-dependent dysfunction and tissue edema. At present, the postulated pathogenesis of I/R injury is associated with the toxic effect of excitatory amino acids (e.g., glutamate), excessive accumulation of oxygen-free radicals and intracellular calcium overload, proinflammatory factor releasing, and especially the production of reactive oxygen species (ROS) [3].

*α*B-crystallin is a prominent member of the small heat-shock protein family, which has been documented to distribute in different tissues such as lens, neural retina, retinal pigment epithelium, heart, skeletal muscle, kidney, and brain [4–6]. As we all know, *α*B-crystallin is not solely recognized in chaperone effect, it also includes antiangiogenesis and antioxidative stress [7–9]. Nowadays, emerging evidences demonstrate that *α*B-crystallin retrieves organs and tissues from the impairment of I/R, including the heart and brain [10, 11]. Nevertheless, there is few research concerning the effect of *α*B-crystallin to protect retina from the damage of I/R.

Considerable reports have revealed that *α*B-crystallin has a neuroprotection in the model of optic nerve crush, which can improve the survival of retinal ganglion cells (RGCs) and function of retina [12–14]. However, there are few studies demonstrating *α*B-crystallin protecting retina from injury of ischemic reperfusion. A most recent investigation has proved that *α*B-crystallin plays a neuroprotective role in retina ischemic reperfusion injury through attenuating the expression of caspase-3 to suppress retina ganglion cell (RGC) death [15]. Furthermore, an increasing number of research has suggested that antioxidant has neuroprotective effect through antioxidation and by modulating NF- (nuclear factor-) kappaB p65 (NF-*κ*B) in retina I/R model [16–18]. The purpose of this study is to investigate whether *α*B-crystallin could prevent retina from I/R injury and further to determine whether this effect is related to antioxidative reaction and downregulation of NF-kappa B.

## 2. Materials and Methods

### 2.1. Animals

The study included 72 adult male Sprague-Dawley rats weighing 180–200 g which were purchased from the Laboratory Animal Center of Chongqing Medical University. Institutional ethics committee at Chongqing Medical University approved all animal experiments. Animal care and all experiments were treated in accordance with the Association for Research in Vision and Ophthalmology (ARVO) guidelines for the Use of Animals in Ophthalmic and Vision Research. The animals were kept in laminar flow racks under a stable temperature and humidity condition with 12-hour light/dark cycles.

### 2.2. Retinal I/R Injury Experiment

Before retinal ischemia reperfusion construction, the animals were anesthetized by an intraperitoneal (i.p.) injection of 3.5% chloral hydrate (1 mL/kg). Topical anesthesia was achieved by using 1 or 2 drops of 0.4% oxybuprocaine hydrochloride (Benoxil, Santen, Japan), and pupillary dilatation was maintained using 0.5% tropicamide (compound tropicamide eye drops, Santen, Japan). Rats were put on a heating blanket to maintain the body temperature at 37°C. After dilation of the pupil, a 27 G needle was inserted into the anterior chamber of the right eye under a microscope, which was connected to a saline reservoir (250 mL). The reservoir was lifted at 150 cm above the eye to increase intraocular pressure (IOP) to 110 mmHg for 60 minutes. Retinal ischemia was confirmed by whitening of the iris and loss of the red reflex of the retina. After 60 minutes, the needle was removed from the anterior chamber and reperfusion was confirmed with observation of iris and fundus vessels. Only the right eye accepted IOP, and ofloxacin ophthalmic gel (0.3%) was applied to the eye through all the experiments. The sham group performed the same procedure except elevating the container.

### 2.3. Drug Administration

The rats were administrated with *α*B-crystallin (Sigma-Aldrich, St. Louis, MO, USA; 1 × 10^−5^ g/L, 5 *μ*L; dissolved in phosphate buffer saline) or vehicle (phosphate buffer saline) via intravitreous injection once immediately after retinal I/R injury. The animals were randomly assigned to three groups, which included sham group, I/R + vehicle group, and I/R + *α*B-crystallin group. Each group had 4–6 rats.

### 2.4. Histopathology

The eyes were enucleated (*n* = 6) at 24 hours, 1 week, and 1 month after I/R injury, respectively, and fixed in 4% paraformaldehyde for 2 hours at 4°C, and anterior segment was removed. The tissues were dehydrated with10% and 20% sucrose, respectively, for an hour and with 30% overnight. Fixed eyeballs were embedded in optimum cutting temperature compound (Sakura, USA) and cut into 10 *μ*m-thick parallel with the maximal circumference of the eye ball through the optic disc. The tissues were stained with hematoxylin and eosin (HE) and observed under light microscope (Leica, Heidelberg, Germany). Light microscope images were obtained at 400x magnification. To evaluate the damage of retinal ischemia reperfusion, the thickness was measured including total retina thickness, the inner plexiform layer (IPL), the inner nuclear layer (INL), and outer nuclear layers (ONL) and the number of cells in the ganglion cell layer (GCL) was calculated using the linear cell density (cells per 200 *μ*m). All measurements were made with 2 to 3 disc diameters from the optic disc, and 3 sections per eye were averaged.

### 2.5. Electroretinogram (ERG)

To access the function of retina at 1 week after I/R, the rats (*n* = 4) were detected with ERG (RETIport32, Roland, Germany). The rats were dark-adapted over 12 hours and anesthetized with sumianxin II. After anesthetization, the cornea was achieved using 1 or 2 drops of 0.4% oxybuprocaine hydrochloride and pupillary dilatation was maintained using 0.5% tropicamide. The rats were placed on a heating blanket to maintain body temperature at 37°C facing the stimulus at a distance of 20 cm. Stainless steel wire loops (0.1 mm diameter) were placed on the center of the cornea, a reference electrode was connected to the middle of the lower eyelid, and a grounding electrode was placed near the tail. Responses to a light flash (2.5 cd · s/m^2^) from a photic stimulator were amplified, and the preamplifier bandwidth was set at 0.3–300 Hz. The amplitude of the a-wave was measured from the baseline to the trough, while that of the b-wave was measured from the maximum of the a-wave trough to the peak of the b-wave. The amplitude of oscillatory potentials (Ops) was measured as the sum of the amplitudes of OP1, OP2, OP3, and OP4.

### 2.6. Determination of the Level of MDA and NO and the Activity of T-SOD

All the eyes (*n* = 6, for each group) were enucleated, and the retina was isolated 24 hours and 1 week after retinal I/R injury. The samples were prepared to 10% homogenized sterile normal saline at the ratio of 1 : 9 according to the weight, and the solution was centrifuged at 2500 rpm for 10 minutes at 4°C. The supernatant was prepared for the following measurements. The 10% homogenates were diluted into 5% for the measurement of malondialdehyde (MDA), and the supernatant was diluted, respectively, into 5% for total superoxide dismutase (T-SOD) and 1% for nitric oxide (NO) and protein concentration. Protein concentrations were detected with the total protein quantitative assay kit (Jiancheng Institute of Biotechnology, Nanjing, China). The levels of MDA were measured by the formation of thiobarbituric acid reactive species with the malondialdehyde assay kit (Jiancheng Institute of Biotechnology, Nanjing, China). The NO was combined with water and oxygen to form nitrates and nitrites whose level indicated the content of NO tested with nitric oxide assay kit (Jiancheng Institute of Biotechnology, Nanjing, China). The T-SOD activity was determined with hydroxylamine assay provided by commercial test kits (Jiancheng Institute of Biotechnology, Nanjing, China). The data were expressed as nanomoles per milligram protein (nM/mgprot) for MDA, micromoles per milligram protein (*μ*mol/mgprot) for NO, and units per milligram protein (U/mgprot) for T-SOD. All of the procedures were performed in ice.

### 2.7. Immunohistochemistry Staining

OCT-embedded retinal sections were put at room temperature for about an hour and washed (three times, 5 minutes) with PBS, incubated with 3% H_2_O_2_ deionized water to make endogenous peroxidase activity inactivate, and blocked the tissue sections in goat serum (Zhongshan Jinqiao Institute of Biotechnology, Beijing, China). Primary antibody inducible nitric oxide synthase (iNOS) (1 : 100; Novus Biological, USA) and NF-kappa B p65 (1 : 300; Biosynthesis Institute of Biotechnology, Beijing, China) were applied to the sections overnight at 4°C, respectively. In the next day, sections were incubated with secondary antibody for 20 minutes at room temperature. Hematoxylin-DAB was used for staining, resulting in a brown signal (Zhongshanjinqiao Institute of Biotechnology, Beijing, China).

### 2.8. Statistical Analysis

All the data were presented as the mean ± SD. Statistical analyses were carried out using SPSS for Windows version 19.0 (SPSS Inc., Chicago, IL). To determine the significant differences, an analysis of variance (ANOVA) was applied. A *p* < 0.05 was considered to be statistically significant.

## 3. Results

### 3.1. *α*B-Crystallin Protects Rat Retina from Histological Damage and RGC Death Caused by I/R Injury

**Figure 1:**
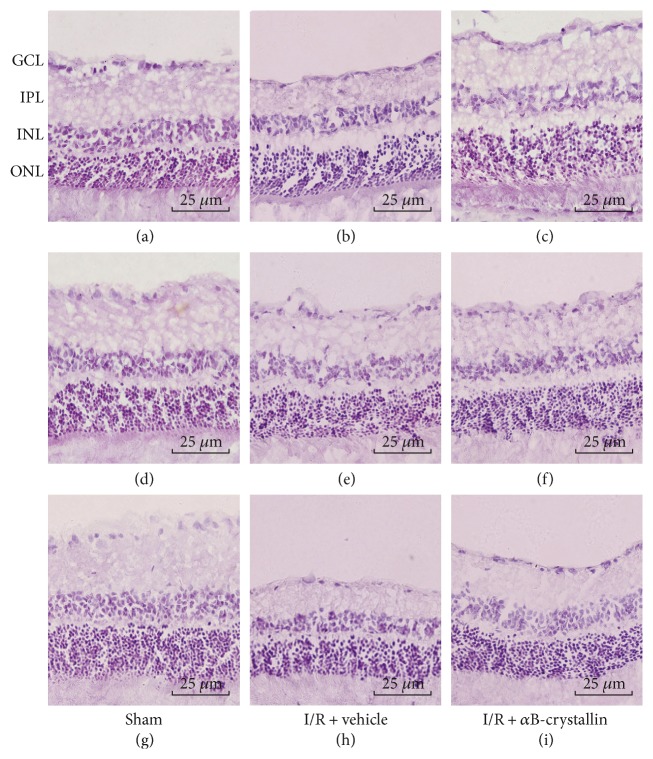
Representative photographs of the rat retina after I/R injury. In the 3 groups (*n* = 6), (a), (b), and (c) are for 24 h; (d), (e), and (f) are for 1 w; and (g), (h), and (i) are for 1 m post I/R. In sham group, the GCL and INL were obvious and well organized. The IPL and INL in I/R + vehicle group was obviously thinner, and the number of RGCs significantly declined, while in I/R + *α*B-crystallin group, the retina was more normal in structure, with a thicker INL than in I/R + vehicle group. GCL: ganglion cell layer; INL: inner nuclear layer; IPL: inner plexiform layer; ONL: outer nuclear layer. Scale bar = 25 *μ*m.

**Figure 2:**
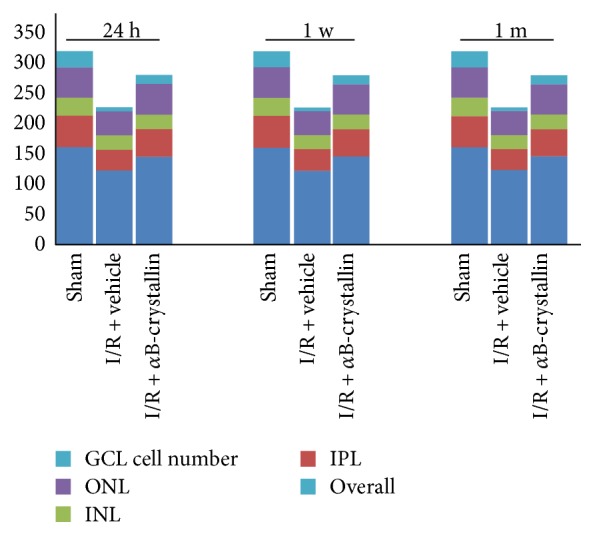
Representative thickness of the rat retina after 24 h and 1 w I/R injury. The results included overall retina, IPL, INL, ONL, and the number of GCL in the 3 groups (*n* = 6). GCL: ganglion cell layer; INL: inner nuclear layer; IPL: inner plexiform layer; ONL: outer nuclear layer.

As shown in Figures 1 and 2, HE-stained retinas were used to access the change of retinal layers at 24 hours, 1 week, and 1 month, respectively, after I/R injury; data was expressed in Table 1. Compared with sham group, the thickness of the retina in I/R ± vehicle group was significantly reduced, which was primarily due to degeneration of cell bodies in the GCL and thinning of the INL, IPL, and ONL. And the changes of rat retinal layers were approximately consistent at 3 time points. The overall retinal thickness in I/R ± vehicle group was reduced by 24%, 24%, and 48%, respectively, compared with the sham group at 24 hours, 1 week, and 1 month after I/R injury (*p* < 0.05). In I/R + vehicle group, the thickness of IPL, INL, and ONL was decreased by 33%, 28%, and 18%, respectively (*p* < 0.05) at 24 hours post-I/R compared with the sham group (*p* < 0.05). In consistent with the change of retinal layers, the thickness of IPL, INL, and ONL in I/R + vehicle group was reduced by 32%, 25%, and 28% at 1 week and 53%, 43%, and 43% at 1 month post-I/R, respectively (*p* < 0.05). The mean density of RGCs in I/R ± vehicle group was obviously lower than that in sham group (7.67 ± 1.51 versus 27.00 ± 1.10, 9.33 ± 2.42 versus 27.67 ± 1.51, and 9.67 ± 1.51 versus 26.67 ± 2.07/200 *μ*m, resp., for 24 hours, 1 week, and 1 month after I/R) (*p* < 0.05).

Compared with I/R + *α*B-crystallin group, the overall retinal thickness in I/R + vehicle group, respectively, was significantly decreased by 16%, 10%, and 38% at 3 points (*p* < 0.05) and the thickness of IPL, INL, and ONL in I/R + vehicle group was reduced by 19%, 13%, and 16% at 24 hour; 18%, 19%, and 15% at 1 week; and 42%, 40%, and 30% at 1 month post-I/R, respectively (*p* < 0.05). The RGCs in I/R + *α*B-crystallin group were significantly greater than those in I/R ± vehicle group (*p* < 0.05).

### 3.2. Protective Effect of *α*B-Crystallin on the Reduction in Amplitude of a-Wave, b-Wave, and OPs

**Figure 3:**
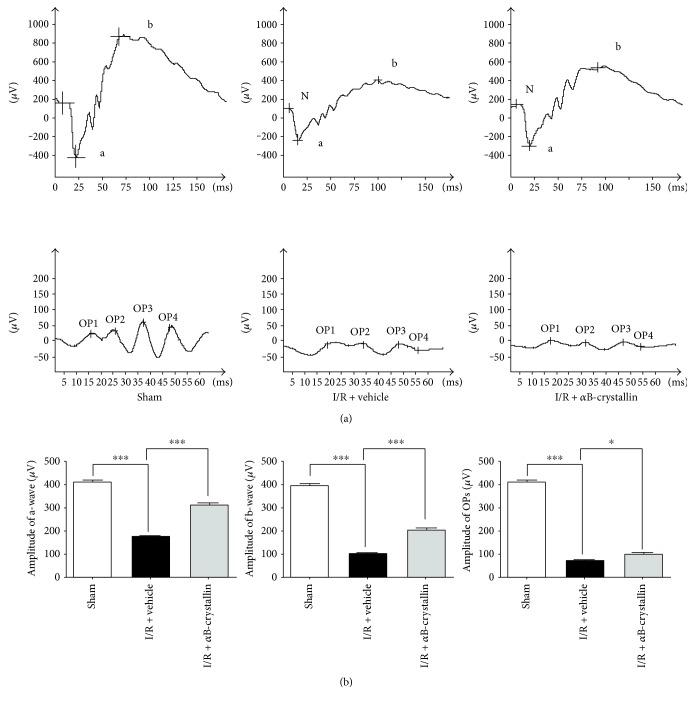
The ERG was measured at 1 w after I/R injury. Representative photographs of the amplitudes of a-wave, b-wave, and OPs were recorded in the three groups (a). Data was expressed as the mean ± SEM. (*n* = 4) (b). ^∗^*p* < 0.05, ^∗∗∗^*p* < 0.001. ERG: electroretinogram; OPs: oscillatory potentials.

To investigate the retina functional changes caused by I/R, ERG was recorded 1 week after I/R.  Figure 3(a) showed the ERG changes for the three groups 1 week after I/R or sham operation; data was shown in Figure 3(b). Compared with sham group, the amplitudes of a-wave, b-wave, and OPs were significantly decreased by 58%, 75%, and 82%, respectively, in I/R + vehicle group (173.50 ± 12.18 versus 409.25 ± 22.04 *μ*V for a-wave, 201.75 ± 16.21 versus 795.00 ± 31.80 *μ*V for b-wave, and 35.51 ± 1.86 versus 204.51 ± 13.16 *μ*V for OPs) (*p* < 0.05). The amplitudes of a-wave, b-wave, and OPs in I/R + *α*B-crystallin group were also lower than those in sham group, but significantly higher than those in I/R + vehicle group (173.50 ± 12.18 versus 312.50 ± 19.96 *μ*V for a-wave, 201.75 ± 16.21 versus 410.00 ± 46.04 *μ*V for b-wave, and 35.51 ± 1.86 versus 49.03 ± 4.54 *μ*V for OPs) (*p* < 0.05).

### 3.3. *α*B-Crystallin Increased the Activity of T-SOD and Inhibited the Level of MDA and NO

**Figure 4:**
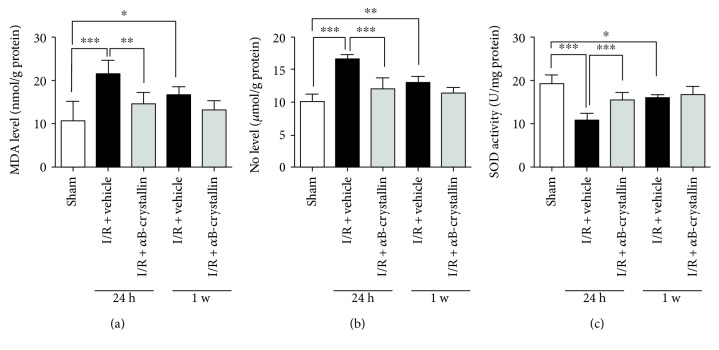
The level of MDA and NO and activities of T-SOD were showed (*n* = 6). The level of MDA and NO was higher in I/R + vehicle and I/R + *α*B-crystallin groups than those in sham group, but was significantly lower in I/R + *α*B-crystallin group than in I/R + vehicle group at 24 h after I/R (*p* < 0.05) (a, b). Activity of T-SOD in I/R + vehicle and I/R + *α*B-crystallin groups became significantly lower than those of sham group at 24 h after I/R (*p* < 0.05); activity of T-SOD in I/R + vehicle group was significantly higher than those in I/R + *α*B-crystallin group at 24 h after I/R (*p* < 0.05) (c). In the content of MDA, NO, and T-SOD, there was no significant difference between I/R + vehicle and I/R + *α*B-crystallin groups (*p* > 0.05) at 1 w ^∗^*p* < 0.05. ^∗∗^*p* < 0.01; ^∗∗∗^*p* < 0.001. I/R: ischemia/reperfusion; MDA: malondialdehyde; NO: nitric oxide T-SOD: total-superoxide dismutase.

To access the effects of *α*B-crystallin on the activity of retinal cellular antioxidants, the cellular levels of MDA, NO, and T-SOD were shown in Figure 4() and data was recorded in Table 2 at 24 hours and 1 week after retinal I/R injury. At 24 hours after I/R injury, the level of MDA was significantly lower in sham group and I/R + *α*B-crystallin group compared with I/R + vehicle group (10.58 ± 4.59, 21.40 ± 3.24, and 14.63 ± 2.58 nmol/mg protein, resp., for sham, I/R + vehicle, and I/R + *α*B-crystallin groups) (*p* < 0.05) (Figure 4(a)). Meanwhile, *α*B-crystallin significantly decreased the level of NO in I/R + *α*B-crystallin group (10.02 ± 1.16, 16.47 ± 0.76, and 11.98 ± 1.68 *μ*mol/g protein for sham, I/R + vehicle, and I/R + *α*B-crystallin groups, resp.) (*p* < 0.05) (Figure 4(b)). We also determined the activity of T-SOD, which was lower in I/R + vehicle group than that in *α*B-crystallin group (46.35 ± 7.96 versus 68.84 ± 7.93 U/mg protein) (*p* < 0.05) (Figure 4(c)). However, the content of MDA, NO, and T-SOD showed no significant difference between I/R + vehicle and I/R + *α*B-crystallin groups (*p* > 0.05) at 1 week, which indicated that *α*B-crystallin can play an antioxidative role at early stage of retinal I/R injury.

### 3.4. *α*B-Crystallin Decreased the Expression of iNOS after I/R Injury

**Figure 5:**
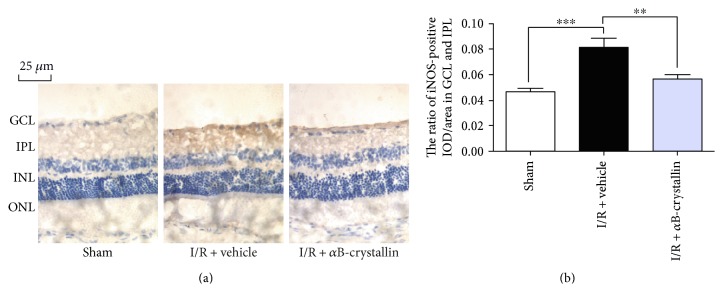
Immunohistochemical staining of iNOS in the rat retina at 1 w after retinal I/R injury. (a) Representative photographs of rat retina section immunohistochemical stained with iNOS. (b) Quantitative analysis of the positive iNOS expression at ratio of IOD/area in the GCL and IPL. GCL: ganglion cell layer; IPL: inner plexiform layer. Data are shown as means ± SD. ^∗∗∗^*p* < 0.001, sham group versus I/R + vehicle group; ^∗∗^*p* < 0.01, I/R + vehicle versus I/R + *α*B-crystallin group. Scale bars = 25 *μ*m.

Immunohistochemical staining of iNOS was measured at 1 week after I/R injury (*n* = 6). Compared with sham group, positive expression of iNOS was dominant in GCL and IPL in I/R + vehicle group and I/R + *α*B-crystallin group (*p* < 0.05). When rats were treated with *α*B-crystallin, the expression of iNOS was less prominent than that in I/R + vehicle group (*p* < 0.05) (Figure 5(a)). The positive expression of iNOS was compared by ratio of IOD/area in GCL and IPL (Figure 5(b)).

### 3.5. *α*B-Crystallin Inhibited the Activation of NF-*κ*B after I/R Injury

**Figure 6:**
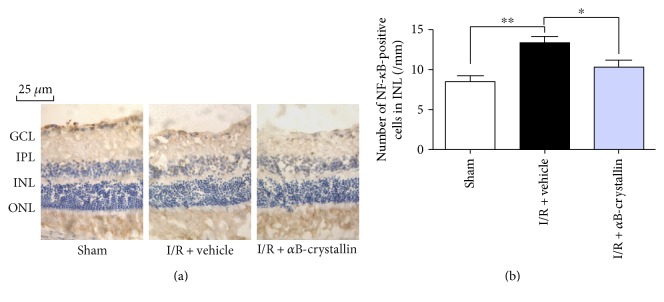
Immunohistochemical staining of NF-*κ*B in the rat retina at 1 w after retinal I/R injury. (a) Representative photographs of rat retina section immunohistochemically stained with NF-*κ*B. (b) Quantitative analysis of the cell number of NF-*κ*B in INL. INL: inner nuclear layer. Data are shown as means ± SD. ^∗∗^*p* < 0.01, sham group versus I/R + vehicle group; ^∗^*p* < 0.05, I/R + vehicle versus I/R + *α*B-crystallin groups. Scale bars = 25 *μ*m.

NF-*κ*B (p65) might be a vital factor for the I/R injury of organs. Immunohistochemistry staining of NF-*κ*B was measured at 1 week after I/R (*n* = 6). The positive NF-*κ*B was expressed in INL, which was significantly observed in I/R + vehicle group compared with I/R + *α*B-crystallin group (*p* < 0.05) (Figure 6(a)). The positive cell number of NF-*κ*B was counted and compared by ratio of IOD/area (Figure 6(b)).

## 4. Discussion

In this study, we revealed that *α*B-crystallin played a neuroprotective role in a rat model with retinal I/R injury. The results showed that *α*B-crystallin could decrease the loss of RGCs and prevent IPL, INL, and ONL from becoming thinner. Compared with I/R + vehicle group, the a-wave, b-wave, and OPs decrement could be reduced by *α*B-crystallin in ERG, which indicated that *α*B-crystallin could decrease the damage of retinal function caused by I/R. Furthermore, *α*B-crystallin showed antioxidant effect by attenuating the level of MDA and NO and increasing the activity of T-SOD. Immunohistochemical staining of retinal sections suggested that *α*B-crystallin could suppress positive expression of iNOS and downregulate activation of NF-*κ*B. These results further demonstrated that *α*B-crystallin played a protective role in retinal ischemia reperfusion through antioxidant and by suppressing the activation of NF-*κ*B.

Our study had demonstrated that there was significantly decrease of RGCs and thickness of retina at 24 hours, 1 week, and 1 month caused by retina I/R, which was corresponded to previous researches [19–22]. And the reduction of IPL and INL thickness were more at early I/R injury stage, which was in good agreement of Ju et al.'s [23] research, and the damage of retinal I/R injury was obvious at 1 month in our study. The induction of cell death within 24 hours of I/R was compatible with the mechanism evoked by the release of ROS [24]. Compared with *α*B-crystallin given, the thickness of retina was more decreasing in I/R + vehicle at 24 hours, 1 week, and 1 month, which indicated that *α*B-crystallin can protect retina from I/R injury at early stage, which may be associated with antioxidation.

ERG was a common and sensitive measurement to evaluate retinal function. The a-waves provided information associated with the photoreceptors, as well as b-waves regarding the physiology of the ONL bipolar and Müller cells [25, 26]. And OPs were triggered by amacrine cells [27]. A diminished amplitude of b-wave had been well known as a poor prognostic sign in retina-ischemic reperfusion [28–30]. And a large number of studies had indicated that there was a remarkably slower recovery of the b-wave in the high IOP model [19–21, 31]. The severe reduction of oscillatory potentials appeared to exist a correlation with a circulatory deficiency in the retina, so the OPs were used to prognose retinal disease, particularly in diabetic retinopathy and ischemia [32]. Whereas these changes were significantly ameliorated by *α*B-crystallin, when administrated with *α*B-crystallin, both a- and b-wave amplitudes were improved. Consequently, we inferred that *α*B-crystallin protected the retina from I/R injury in functional morphology. But it was not clear how *α*B-crystallin played a protective effect in retinal ischemia reperfusion.

Many studies have revealed irreversible cellular damages caused by retinal I/R. At cellular level, ischemic retinal injury consists of energy failure, glutamate excitability toxicity, calcium overload, inflammation, and oxidative stress, which lead to cell necrosis or apoptosis. It is well known that oxidative stress plays a vital role in I/R injury. MDA is a naturally degraded product of lipid peroxidation, a process which can react with the amino group of nucleic acids to produce cytotoxicity when unsaturated fat-soluble substances (lipids) are oxidized to form radicals [33]. NO is an important neuromediator implicated in many physiological processes in the retina which reacts with superoxide to form peroxynitrite. As a strong oxidant, peroxynitrite causes lipid peroxidation, which leads to DNA damage and makes SOD inactivated [34]. Therefore, the level of MDA and NO is taken as markers of the severity of cellular damage. Meanwhile, the balance of oxidants and antioxidants (e.g., T-SOD and GSH) is responsible for cellular homeostasis [35] and the activity of T-SOD is an indicator for evaluating antioxidant enzyme status. Therefore, the changes of MDA, NO, and T-SOD can reflect the damage of retina caused by oxidative stress during retinal I/R injury.

It has been reported that *α*B-crystallin belongs to the small heat-shock protein (sHSP) superfamily. In addition to their chaperone functions, several studies have proved that the effects of *α*B-crystallin are involved in anti-inflammatory, antioxidation, antiapoptosis, and antiangiogenesis [7–9]. In vitro study, it has shown that *α*B-crystallin treatment not only suppresses the increase in lipid peroxidation levels but also inhibits the lipid breakdown resulting from auto-oxidation by increasing the activities of SOD in mouse cerebral cortex homogenate [36]. In Romi et al.'s research, *α*B-crystallin modulates superoxide dismutase-1 (SOD-1) tissue accumulation in familial amyotrophic lateral sclerosis [37]. As known in Huang et al.'s reports, *α*B-crystallin might be important for myocardial protection during the early phase of ischemic preconditioning, which may associate with attenuating the production of MDA [38]. Wu et al. had suggested that intravenous injection of *α*B-crystallin could be a possible strategy for the treatment of optic nerve injury by inhibiting TNF-alpha and iNOS protein expression, and iNOS was a subunit of NO [39]. In addition, the significant antioxidation of *α*B-crystallin had been investigated in retinal pigment epithelium (RPE) by Kannan et al. [9]. Mueller et al. revealed that increasing levels of alpha-crystallin were found in the lens and retina following intravitreal injection of homo- and hetero-oligomers in rats [40]. Besides, accumulating evidences have suggested the neuroprotective effect of exogenous *α*B-crystallin in the model of rat optic nerve crush [41–43]. There were two ways [42, 43] of *α*B-crystallin given to rats through intravitreal (1 × 10^−5^ g/L, 5 *μ*L for once) or intravenous injection (50 *μ*g/100 g for 10 times), but no evidence to determine the superiority between them. With regard to the content and given times of *α*B-crystallin and safety, we determined its protection in rat I/R injury with 5 *μ*L (1 × 10^−5^ g/L) intravitreously. As reported in our study, the level of MDA and NO significantly reduced and the activity of T-SOD obviously increased when treated with *α*B-crystallin after retina I/R injury, which indicated that *α*B-crystallin had an antioxidative effect.

NF-*κ*B, a redox-sensitive transcription factor, plays a critical role in neuronal cell death in retinal I/R injury [18]. And the expression of NF-*κ*B is likely to be instrumental in the upregulation of iNOS, which expresses increasingly in inner retinal layer when subjected to I/R [44]. Recent studies have demonstrated that protecting the retina from ischemia-reperfusion injury can be done by reducing the expression of NF-*κ*B [16–18]. And other researches have suggested that increasing expression of *α*B-crystallin and suppressing activity of NF-*κ*B can play a role in inhibiting apoptosis or neuroinflammation [45, 46]. However, Adhikari et al. demonstrated that phosphorylation of *α*B-crystallin upregulated the expression of NF-*κ*B to exert its antiapoptosis to protect myoblasts from cytotoxicity when treated with TNF-*α* [47]. Mercatelli et al. also revealed that skeletal myoblasts could be protected by the activating NF-*κ*B and/or overexpressing *α*B-crystallin involved in oxidative stress [48]. Then, *α*B-crystallin directly resists tissue damages by mitigating or activating the expression of NF-*κ*B. In this study, we showed that administration with *α*B-crystallin diminished the positive expression of iNOS and NF-*κ*B after I/R. Consequently, the finding clarified that *α*B-crystallin could alleviate NF-*κ*B expression by reducing the amount of iNOS to attenuate the severity of damage induced by I/R.

## 5. Conclusion

We have demonstrated that *α*B-crystallin had a neuroprotective effect on the retina after ischemia reperfusion injury through its antioxidant activities and achieved by inhibiting the expression of NF-*κ*B. But our study has some limitations. First, *α*B-crystallin was administered after the induction of ischemia reperfusion once immediately, which would not indicate the effective period. Second, we only detected that *α*B-crystallin protected the retina from I/R injury through antioxidant and the signal pathway of NF-*κ*B, but there are further signal pathways that deserved to be investigated. Generally, our available data indicate that *α*B-crystallin can be a potential therapeutic drug for retinal I/R-related diseases.

## Figures and Tables

**Table 1:** Thickness of the retinal layers and GCL cell count at 24 h, 1 w, and 1 m after I/R ($\overset{-}{x}\pm s$).

Group	Thickness (*μ*m)				GCL cell number
Each *N* = 6	Overall	IPL	INL	ONL	(200 *μ*m)
Sham	158.84 ± 3.75	52.86 ± 2.72	29.66 ± 1.99	49.80 ± 1.42	27.00 ± 1.10
24 h I/R + vehicle	121.10 ± 3.03^a,^^∗^	35.62 ± 2.92^a,^^∗^	21.38 ± 1.13^a,^^∗^	40.56 ± 0.98^a,^^∗^	7.67 ± 1.51^a,^^∗^
24 h I/R + *α*B	144.05 ± 2.55^b,^^∗^	45.07 ± 1.68^b,^^∗^	24.46 ± 1.61^b,^^∗^	48.56 ± 2.66^b,^^∗^	15.67 ± 1.97^b,^^∗^
1 w I/R + vehicle	120.32 ± 2.14^a,^^∗^	36.06 ± 1.41^a,^^∗^	22.33 ± 0.93^a,^^∗^	35.89 ± 2.15^a,^^∗^	9.33 ± 2.42^a,^^∗^
1 w I/R + *α*B	134.03 ± 1.10^b,^^∗^	43.98 ± 0.49^b,^^∗^	27.68 ± 0.72^b,^^∗^	42.42 ± 1.41^b,^^∗^	15.00 ± 2.10^b,^^∗^
1 m I/R + vehicle	82.83 ± 4.14^a,^^∗^	24.67 ± 1.72^a,^^∗^	16.77 ± 1.12^a,^^∗^	28.23 ± 3.53^a,^^∗^	9.67 ± 1.51^a,^^∗^
1 m I/R + *α*B	133.49 ± 1.69^b,^^∗^	42.31 ± 1.41^b,^^∗^	27.91 ± 0.67^b,^^∗^	40.26 ± 2.71^b,^^∗^	17.33 ± 1.63^b,^^∗^

^a^Compared with sham group. ^b^Compared with I/R + vehicle; ^∗^*p* < 0.05. INL: inner nuclear layer; IPL: inner plexiform layer; ORL: outer retinal layer; GCL: ganglion cell layer; I/R: ischemia-reperfusion.

**Table 2:** Effect of *α*B-crystallin on the activity of SOD and the level of MDA and NO after I/R ($\overset{-}{x}\pm s$).

Group	MDA (nM/mgprot)	NO (*μ*mol/mgprot)	T-SOD (U/mgprot)
Sham	10.58 ± 4.59	10.02 ± 1.16	85.78 ± 9.57
24 h I/R + vehicle	21.40 ± 3.24^a,^^∗^	16.47 ± 0.76^a,^^∗^	46.35 ± 7.96^a,^^∗^
24 h I/R + *α*B-crystallin	14.63 ± 2.58^b,^^∗^	11.98 ± 1.68^b,^^∗^	68.84 ± 7.93^b,^^∗^
1 w I/R + vehicle	16.55 ± 1.99^a,^^∗^	11.27 ± 2.05^a,^^∗^	70.05 ± 3.98^a,^^∗^
1 w I/R + *α*B-crystallin	13.11 ± 2.21	11.26 ± 0.99	73.96 ± 9.05

^a^Compared with sham group. ^b^Compared with I/R + vehicle. ^∗^*p* < 0.05, each *n* = 6.
